# Dataset for modeling Beck’s cognitive triad to understand depression

**DOI:** 10.1016/j.dib.2021.107431

**Published:** 2021-09-25

**Authors:** Shreekant Jere, Annapurna P. Patil, Ganeshayya I. Shidaganti, Shweta S. Aladakatti, Laxmi Jayannavar

**Affiliations:** a MS Ramaiah Institute of Technology, Bengaluru, India Affiliated to VTU, Belagavi, India; b Dayananda Sagar University, Bengaluru, India

**Keywords:** Cognitive triad, Depression, Sentiment classification

## Abstract

This article presents data to model Beck’s cognitive triad to understand the subjective symptoms of depression, such as negative view of self, future, and world. The Cognitive Triad Dataset (CTD) comprises 5886 messages, 600 from the Time-to-Change blog, 580 from Beyond Blue personal stories, and 4706 from Twitter. The data were manually labeled by skilled annotators. This data is divided into six categories: self-positive, world-positive, future-positive, self-negative, world-negative, and future-negative. The Cognitive Triad Dataset was evaluated on two subtasks: aspect detection and sentiment classification on given aspects. The dataset will aid in the comprehension of Beck’s Cognitive Triad Inventory (CTI) items in a person’s social media posts.

## Specifications Table

**Table:** 

Subject	Health psychology
Specific subject area	Beck’s cognitive theory
Type of data	Text
How data was acquired	The data from Tweeter was extracted using the Twitter API. Data from the Time-to-Change blog and Beyond Blue personal stories are manually collected.
Data format	Raw and analyzed.
Parameters for data collection	The Tweeter API was utilized to capture tweets using filter keywords related to cognitive triad aspects. The keywords related to self, future, and world include {“I”, “myself”, “me”}, {“future”, “from now”, “look forward”, “turn out”, “am going to”, “are going to”, “won’t”, “will”}, and {“world”, “globe”, “people”, “he”, “she”, “it”, “they”, “nobody”, “others”, “obstacle”} respectively.
Description of data collection	The data from Tweeter was extracted using the Twitter API. The filter keywords related to cognitive triad aspects were used in the Tweeter API to capture tweets. The data from the Time-to-Change blog were manually collected. The GitHub code was used to generate simulated data that resembles cognitive patterns found in the Beyond Blue personal stories. The data were manually labeled by skilled annotators. The data includes messages from 798 adult Tweeters and 42 adult Time-to-Change blog users from all over the world.
Experimental factors	Data were preprocessed by deleting duplicate Tweets, incomplete Tweets, and Tweets shorter than four words, removing punctuations and stop words from the text, and deconstructing multi-word hashtags into individual words.
Data source location	Tweeter, Time-to-Change blog and Beyond Blue personal stories.
Data accessibility	Raw data can be retrieved from the Mendeley repository https://data.mendeley.com/datasets/wb2n39sgbp/1[1]. The source code is available online at https://github.com/bctriad/code.

## Value of the Data

• Patients may under- or over-report their symptoms during traditional clinical interviews, depending on the actual or perceived implications for a mental health disorder diagnosis. Intelligent mental disorder understanding systems trained with CTD can overcome these limitations and effectively test for depression.
• The CTD presents 6-ary cognitive triad labels to understand the CTI-items associated with statements in a person’s social media messages. 6-ary labels include self-negative, future-negative, world-negative, self-positive, future-positive, and world-positive.
• The data can be utilized to train a sentiment analysis model, which can then be used for initial screening of depression based on the client’s recent interactions with the clinical chatbot or their social media data.
• The labeled text data can be used to train machine learning models for sentiment analysis and aspect detection tasks. The aspect-based sentiment classification model on CTD can assist psychologists in identifying the cognitive triad aspect-sentiment pairs {(self, negative), (world, negative), (future, negative)} from the social media messages of the individual.

## 1 Data Description

Beck [2] determined three factors responsible for depression: faulty information processing (errors in logic), cognitive triad (negative thinking about world, self, and future), and negative self-schemas. Critical evaluation of Beck’s theory is provided in Alloy et al. [3] and Butler et al. [4]. This section highlights the cognitive triad which, can be modeled using sentiment analysis. The Cognitive Triad Inventory (CTI) comprises items [5] related to a view of the self, the world, and the future, as shown in Table 1.

**Table 1:** CTI items and expected answers from depressed person.

CTI items	Aspect (Subscale) self, world, future	Expected answer from depressed person (Binary Yes/No class)	Expected answer from depressed person with Scale 1–7 (Higher scores indicate true views and lower scores indicate false views)
Everything in the future will turn out perfectly for me.	Future	No	False view
I believe some things are going really well for me a couple of years from now.	Future	No	False view
I can do many things right.	Self	No	False view
I think when I grow older, I will be happy.	Future	No	False view
I value myself.	Self	No	False view
A lot of good things are going to happen to me in the future.	Future	No	False view
I do a lot of different things well.	Self	No	False view
I want to think about the positive stuff that is going to happen to me in the future.	Future	No	False view
I am a loser.	Self	Yes	True view
I dislike myself.	Self	Yes	True view
I am a really good person.	Self	No	False view
I do my schoolwork properly.	Self	No	False view
I will fix my issues.	Future	No	False view
There is nothing left in my life that I can look forward to.	Future	Yes	True view
No matter what happens to me, my family doesn’t care.	World	Yes	True view
My worries and problems will never go away.	Future	Yes	True view
I am faced with several obstacles.	World	Yes	True view
Lots of bad things happen to me.	World	Yes	True view
I feel guilty of several things.	Self	Yes	True view
I have personality issues.	Self	Yes	True view

**Fig. 1:**
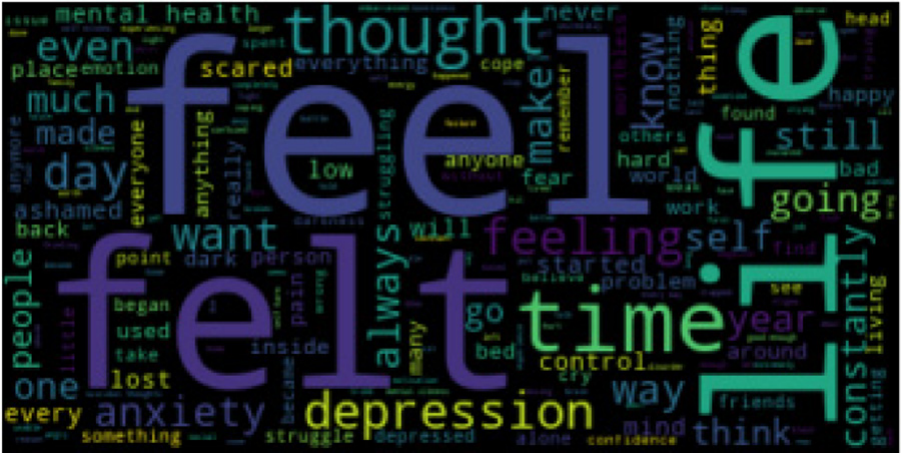
Word cloud for self-negative label.

**Fig. 2:**
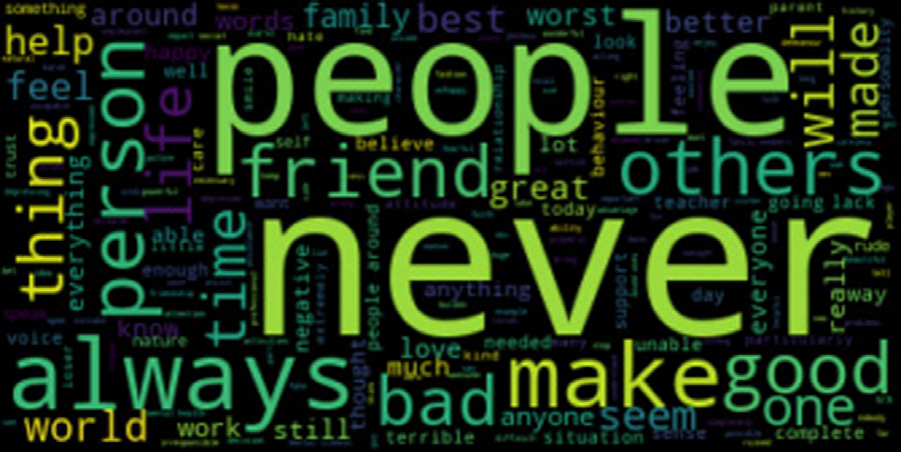
Word cloud for world-negative label.

**Fig. 3:**
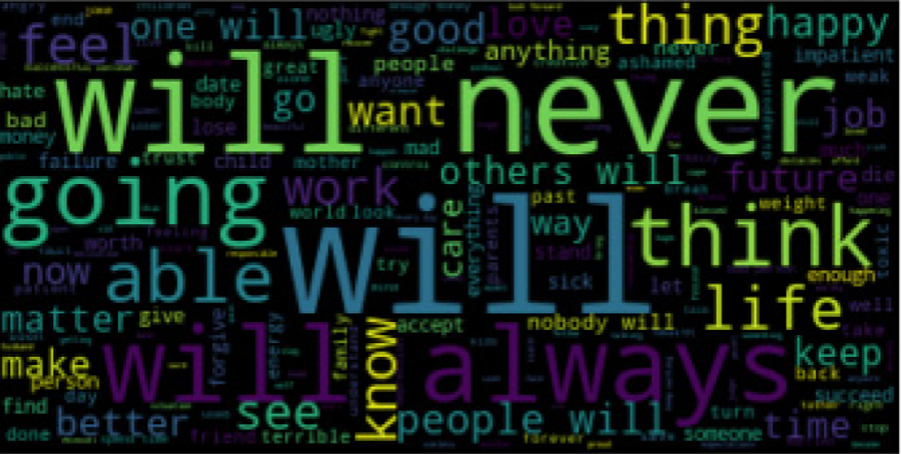
Word cloud for future-negative label.

**Fig. 4:**
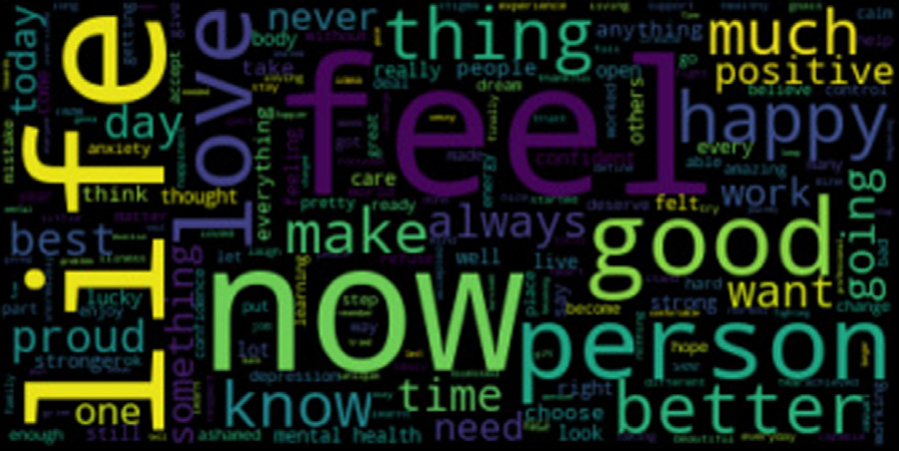
Word cloud for self-positive label.

**Fig. 5:**
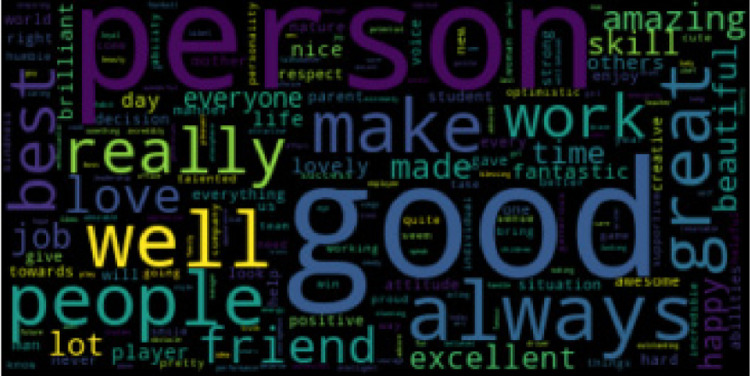
Word cloud for world-positive label.

**Fig. 6:**
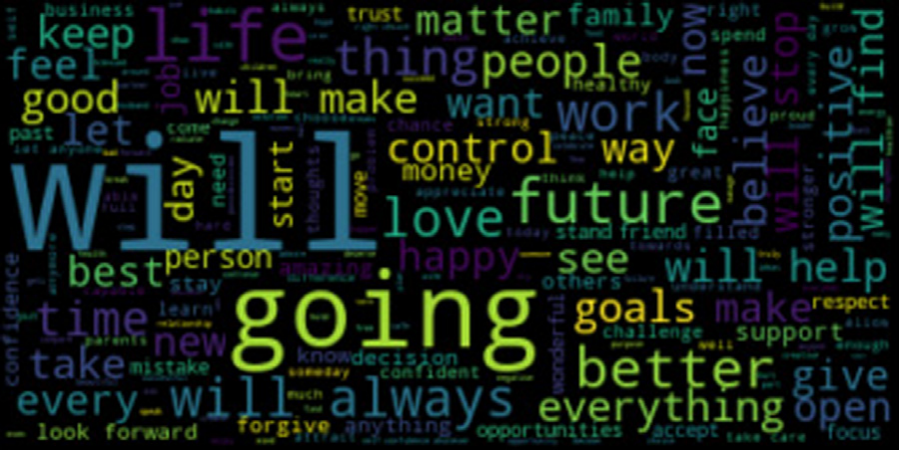
Word cloud for future-positive label.

The Cognitive Triad Dataset is used to understand the CTI-items associated statements in a person’s social media messages. 6-ary classes include C6 = {self-negative (sneg), world-negative (wneg), future-negative (fneg), self-positive (spos), world-positive (wpos), future-positive (fpos)}. We collected data from Tweeter, Time-to-Change blog, and Beyond Blue personal stories and used the majority vote for our dataset with the gold standard. The statistics for the 6-ary dataset is provided in Table 2. For cognitive aspect detection, CTD classes are reduced to ternary classes {self, world, future}. CTD statistics for cognitive aspects are given in Table 3. For sentiment classification, CTD classes are decreased to binary classes {positive, negative}. Table 4 shows the CTD statistics for sentiment classification. Word clouds for self-negative, world-negative, future-negative, self-positive, world-positive, and future-positive labels are provided in Fig. 1, Fig. 2, Fig. 3, Fig. 4, Fig. 5, Fig. 6. A word cloud is a depiction of text data in which the size of each word signifies its frequency or relevance.

**Table 2:** 6-ary CTD statistics.

Corpus	sneg	wneg	fneg	spos	wpos	fpos
Tweeter	797	768	784	793	787	777
Time to Change	106	102	103	102	90	97
Beyond Blue	95	90	93	97	107	98
Total	998	960	980	992	984	972

**Table 3:** CTD statistics on cognitive aspects.

Corpus	Self	World	Future
Tweeter	1590	1555	1561
Time to Change	208	192	200
Beyond Blue	192	197	191
Total	1990	1944	1952

**Table 4:** CTD statistics on cognitive sentiments.

Corpus	Negative	Positive
Tweeter	2349	2357
Time to Change	311	289
Beyond Blue	278	302
Total	2938	2948

## 2 Experimental Design, Materials and Methods

The cognitive triad dataset is evaluated for aspect detection and sentiment classification using popular machine learning and deep learning models. Data were preprocessed by deleting duplicate Tweets, incomplete Tweets, and Tweets shorter than four words, removing punctuations and stop words from the text, and deconstructing multi-word hashtags into individual words. In the preliminary work, Decision Tree, Random Forest, Naive Bayes, SVM [6], and RNN-Capsule [7] models are evaluated for aspect extraction and sentiment classification on the cognitive triad dataset. The baseline machine learning models are implemented using scikit-learn. The RNN-capsule model is implemented using PyTorch and run on a single GPU (NVIDIA GeForce RTX 3080 Ti). By default, we trained the model for 28 epochs with a batch size of 32. We employed pre-trained GloVe for the word embedding. In numerous trials, we chose the best validation performance and presented the testing performance in experimental results. Table 5 compares various models on CTD for aspect extraction task. The results of accuracy and an F1-score are very close for Random Forest and Support Vector Machine. The RNN Capsule model has a maximum accuracy of 96.17% and an F1-score of 96.02%. Table 6 provides the comparison of various models on CTD for the sentiment classification task. The results of accuracy and F1-score are very close for Decision Tree and Support Vector Machine. The Random Forest model has the highest accuracy of 81.58% and an F1-score of 81.56% among machine learning models. The RNN Capsule model has a maximum accuracy of 88.87% and an F1-score of 88.55% for the sentiment classification task. Table 7 gives the performance of various models on CTD for sentiment classification task on the self aspect. The results of accuracy and F1-score are very close for Random Forest and Support Vector Machine. The RNN Capsule model has a maximum accuracy of 83.67% and an F1-score of 83.72% for the sentiment classification task on the self aspect. Table 8 provides the performance of various models on CTD for sentiment classification task on the future aspect. The Random Forest model has the highest accuracy of 83.62% and an F1-score of 84.11% among machine learning models. The RNN Capsule model has a maximum accuracy of 90.06% and an F1-score of 89.89% for the sentiment classification task on the future aspect. Table 9 gives the performance of various models on CTD for sentiment classification task on the world aspect. The Random Forest model has the maximum accuracy of 86.60% and an F1-score of 86.59% for the sentiment classification task on the world aspect. Table 10 provides the performance of aspect based sentiment classification on cognitive 〈aspect, sentiment〉 classes. The Support Vector Machine has the highest accuracy of 60.54% and an F1-score of 60.58% among machine learning models. The RNN Capsule model has a maximum accuracy of 85.71% and an F1-score of 85.84% for the sentiment classification task.

**Table 5:** Performance of aspect extraction on CTD.

Model	Accuracy	Precision	Recall	F1-score
Decision Tree	70.25	70.28	70.42	70.35
Random Forest	76.58	76.65	76.74	76.69
Naive Bayes	54.33	61.77	54.18	57.73
Support Vector Machine	77.25	77.84	77.35	77.59
RNN-Capsule	96.17	96.86	95.20	96.02

**Table 6:** Performance of sentiment classification on CTD.

Model	Accuracy	Precision	Recall	F1-score
Decision Tree	76.25	76.29	76.14	76.21
Random Forest	81.58	81.61	81.51	81.56
Naive Bayes	64.83	70.31	65.65	67.90
Support Vector Machine	77.83	79.03	78.16	78.59
RNN-Capsule	88.87	89.62	87.50	88.55

**Table 7:** Performance of sentiment classification on self aspect.

Model	Accuracy	Precision	Recall	F1-score
Decision Tree	73.11	73.12	73.00	73.06
Random Forest	77.13	77.27	76.97	77.12
Naive Bayes	67.08	69.89	66.36	68.08
Support Vector Machine	75.38	76.55	74.97	75.75
RNN-Capsule	83.67	83.44	84.00	83.72

**Table 8:** Performance of sentiment classification on future aspect.

Model	Accuracy	Precision	Recall	F1-score
Decision Tree	81.88	82.18	82.01	82.09
Random Forest	83.62	84.40	83.83	84.11
Naive Bayes	68.73	76.59	69.46	72.85
Support Vector Machine	80.40	81.49	80.65	80.07
RNN-Capsule	90.06	90.28	89.04	89.89

**Table 9:** Performance of sentiment classification on world aspect.

Model	Accuracy	Precision	Recall	F1-score
Decision Tree	79.65	80.03	79.80	79.91
Random Forest	86.60	86.60	86.58	86.59
Naive Bayes	69.73	76.56	69.01	72.59
Support Vector Machine	80.89	81.68	80.67	81.17
RNN-Capsule	86.05	86.80	84.46	85.61

**Table 10:** Performance of aspect based sentiment classification on cognitive 〈aspect, sentiment〉 classes.

Model	Accuracy	Precision	Recall	F1-score
Decision Tree	52.65	53.31	52.65	52.62
Random Forest	58.64	59.05	58.52	58.26
Naive Bayes	44.65	46.75	44.15	42.17
Support Vector Machine	60.54	61.69	60.35	60.58
RNN-Capsule	85.71	85.99	85.69	85.84

## Ethics Statement

The data presented in this article is being distributed in accordance with the Twitter developer policy (https://developer.twitter.com/en/developer-terms/policy), Beyond Blue terms of use (https://www.beyondblue.org.au/general/terms-of-use), and Time-to-Change privacy policy (https://www.time-to-change.org.uk/privacy-policy).

## CRediT authorship contribution statement

**Shreekant Jere:** Conceptualization, Methodology, Data curation, Investigation, Writing – original draft. **Annapurna P. Patil:** Investigation. **Ganeshayya I. Shidaganti:** Writing – original draft. **Shweta S. Aladakatti:** Writing – review & editing. **Laxmi Jayannavar:** Investigation.

## Declaration of Competing Interest

The authors declare that they have no known competing financial interests or personal relationships that could have appeared to influence the work reported in this paper.
